# Effects of high-intensity exercise on rehabilitation of patients after stroke: a systematic review and meta-analysis of randomized controlled trials with high quality

**DOI:** 10.3389/fneur.2025.1565118

**Published:** 2025-08-06

**Authors:** Shuang Li, Yuchang Dou, Hong Li

**Affiliations:** ^1^Department of Neurology, China-Japan Union Hospital of Jilin University, Changchun, China; ^2^Department of Traditional Chinese Medicine, China-Japan Union Hospital of Jilin University, Changchun, China; ^3^Department of Nursing, China-Japan Union Hospital of Jilin University, Changchun, China

**Keywords:** high-intensity, exercise, stroke, randomized controlled trials, meta-analysis

## Abstract

**Objectives:**

To present the latest systematic review and meta-analysis of high-quality randomized controlled trials (RCTs) comparing high-intensity exercise with routine rehabilitation in stroke patients.

**Methods:**

PubMed, Web of Science, and Cochrane were used to searching literature up to October 2024. RCTs with sample size of ≥50 individuals were included. Primary outcomes assessed were the Six-Minute Walking Test (6MWT), Ten-Meter Walk Test (10MWT), VO_2peak_, Berg Balance Scale (BBS), Timed Up and Go test (TUG), and Montreal Cognitive Assessment (MoCA). Standardized mean differences (SMD) with 95% confidence intervals (CI) were used for pooling data. Stability was evaluated by sensitivity analysis.

**Results:**

Seven RCTs with 724 participants were included. Meta-analysis revealed significant improvements in the 6MWT (SMD: 0.28; 95% CI: 0.11, 0.45) and BBS (SMD: 0.35; 95% CI: 0.03, 0.67) in the high-intensity exercise group. However, high-intensity exercise had no significant effect on VO_2peak_, TUG, or 10MWT. Sensitivity analysis showed that all outcomes were stable except for the 10MWT. No significant publication bias was detected for any indicator.

**Conclusion:**

High-intensity exercise significantly improves 6MWT and BBS in stroke patients, but does not significantly affect TUG, VO_2peak_, 10MWT, or MoCA. Clinicians should encourage stroke patients with walking function to engage in structured high-intensity exercise to improve cardiopulmonary function.

**Systematic review registration:**

CRD42024623036 Publicly accessible URL: https://www.crd.york.ac.uk/PROSPERO/view/CRD42024623036.

## 1 Introduction

Stroke has become a major global public health issue. The Global Burden of Disease report indicates that the stroke’ incidence in China is higher than in other developed countries and continues to rise, with the burden of stroke-related diseases growing increasingly severe (1). Motor dysfunction is a leading cause of disability among stroke survivors (2). Statistics show that over 80% of stroke survivors experience varying degrees of motor function loss, and 50% still have motor dysfunction 3 months after the stroke (3, 4). Studies have shown that, compared to age-and gender-matched healthy individuals, stroke patients have about 50% reduced cardiopulmonary fitness in the early stages (5). In some patients, this cardiopulmonary fitness continues to deteriorate, significantly increasing the risk of cardiovascular disease. Improved cardiopulmonary reserve can optimize peripheral muscle oxygen supply and promote limb function recovery by improving metabolic efficiency, thereby improving quality of life and reducing the risk of recurrence (6, 7). Therefore, improving the cardiopulmonary reserve in stroke patients is crucial for enhancing limb function, improving quality of life, and preventing disease recurrence. Exercise interventions, as a key rehabilitation method, play a vital role in promoting the recovery of daily living abilities and reintegration into family and society for stroke survivors (8).

Currently, two common exercise intervention approaches are used to improve mobility and aerobic capacity: moderate-intensity training and high-intensity training. Moderate-intensity training is typically based on moderate-intensity continuous training (MICT), which involves continuous exercise at 64 to 75% of the maximum heart rate (HR_max_) or 40 to 59% of the heart rate reserve (HRR). On the other hand, the most common form of high-intensity training is high-intensity interval training (HIIT), which alternates between short bursts of intense activity and longer recovery periods (9). Studies have shown that HIIT is more effective than low-and moderate-intensity exercise in improving aerobic capacity and other functions in healthy adults and patients with cardiovascular diseases (10–13). In addition, studies have shown that HIIT also has significant advantages in improving executive function and metabolic regulation (14, 15). Due to safety considerations, The American Heart Association now recommends moderate exercise for stroke patients to enhance their exercise volume and aerobic fitness (16, 17). However, numerous studies have demonstrated that high-intensity exercise offers greater benefits than low-and moderate-intensity exercise for stroke rehabilitation (18–20).

The meta-analysis by Baricich et al. (21), which included randomized controlled trials (RCTs) published before May 2023, found that high-intensity exercise significantly improved the Six-Minute Walking Test (6MWT) and VO_2peak_ in stroke patients. However, no significant differences were observed in the Berg Balance Scale (BBS), Ten-Meter Walk Test (10MWT), or Timed Up and Go test (TUG). This meta-analysis, however, had several limitations, including a high proportion of small-sample studies, which introduced significant heterogeneity and potential small sample size effects that reduced the quality of evidence. Moreover, several recent well-designed, large-sample RCTs have reported the rehabilitation effects of high-intensity exercise on stroke patients, potentially altering the current understanding of its role in post-stroke rehabilitation (22–26). Therefore, this article aims to conduct an updated meta-analysis of high-quality RCTs to more precisely evaluate the effects of high-intensity exercise on post-stroke rehabilitation.

## 2 Methods

### 2.1 Literature search

According to the PRISMA 2020 guidelines (Preferred Reporting Items for Systematic Reviews and Meta-Analyses), meta-analysis was conducted (27) and registered with PROSPERO (CRD42024623036). Literature searching was performed via PubMed, Embase, Web of Science, and Cochrane up to October 2024 for randomized controlled trials (RCTs) comparing high-intensity exercise with routine rehabilitation for stroke patients. The search terms included “exercise,” “stroke,” and “high-intensity.” Additionally, we manually screened the reference lists of all included RCTs. Two authors independently retrieved and assessed eligible articles. Disagreements in the literature selection were resolved through discussion. The full literature search strategy is provided in Supplementary Table S1.

### 2.2 Inclusion and exclusion criteria

#### 2.2.1 Eligibility criteria

P (Population): The study subjects were patients diagnosed with stroke by imaging.
I (Intervention): High-intensity exercise is the intervention method that this study focuses on, usually used alone or in combination with other conventional rehabilitation.
C (Comparison): Control measures included routine rehabilitation, including moderate or low-intensity exercise, or no exercise intervention.
(Outcomes): The main outcome measures including 6MWT, 10MWT, VO_2peak_, TUG, BBS, MoCA, etc.
S (Study design): To minimize the effect of small sample size, only randomized controlled trials (RCTs) with a total sample size ≥50 participants were included.

#### 2.2.2 Exclusion criteria

Study protocols; unpublished studies; non-original studies (including meeting abstracts, corrections, and replies); non-RCT studies; studies without sufficient data; reviews; RCTs with sample size of less than 50 participants.

### 2.3 Data abstraction

Two researchers conducted data abstraction independently, with any differences resolved by a third author. The following information was abstracted from eligible RCTs: first author name, publication year, research period, study region, study design, registration number, intervention, control, sample size, age, gender, intervention time, 6MWT, 10MWT, VO_2peak_, TUG, BBS, and MoCA (because cognitive impairment is a common sequela of stroke, MoCA is included to evaluate the impact of intervention on cognitive function). If research data were insufficient, corresponding authors were contacted for complete data when available.

### 2.4 Quality evaluation

Cochrane Handbook for Systematic Reviews of Interventions 5.1.0 was used to assess the RCTs’ quality, focusing on seven domains: sequence generation, allocation concealment, blinding of participants and personnel, outcome assessment blinding, incomplete outcome data, selective outcome reporting, and other potential biases (28). Each domain was rated as low, high, or unclear risk. Studies with more low-risk evaluations were considered of higher quality. Two authors independently assessed the quality, resolving disagreements through discussion.

### 2.5 Statistical analysis

Review Manager 5.4.1 was used for performing data synthesis. For continuous outcomes, standardized mean differences (SMD) with 95% confidence intervals (CI) were calculated. Chi-squared (*χ*^2^) test (Cochran’s *Q*) and the inconsistency index was conducted to assess heterogeneity (29). Substantial heterogeneity was defined as a *χ*^2^ *p*-value <0.1 or *I*^2^ > 50%. The overall SMD was computed using the random-effects model. For outcomes with more than two studies, sensitivity analysis with one-by-one exclusion was conducted to assess the impact of each individual RCT on the overall result. Funnel plots and Egger’s regression test was conducted for evaluating publication bias (30) in Stata 15.1 (Stata Corp, College Station, Texas, United States). A *p*-value <0.05 was considered indicative of significant publication bias.

## 3 Results

### 3.1 Literature retrieval, study characteristics, and baseline

**Figure 1:**
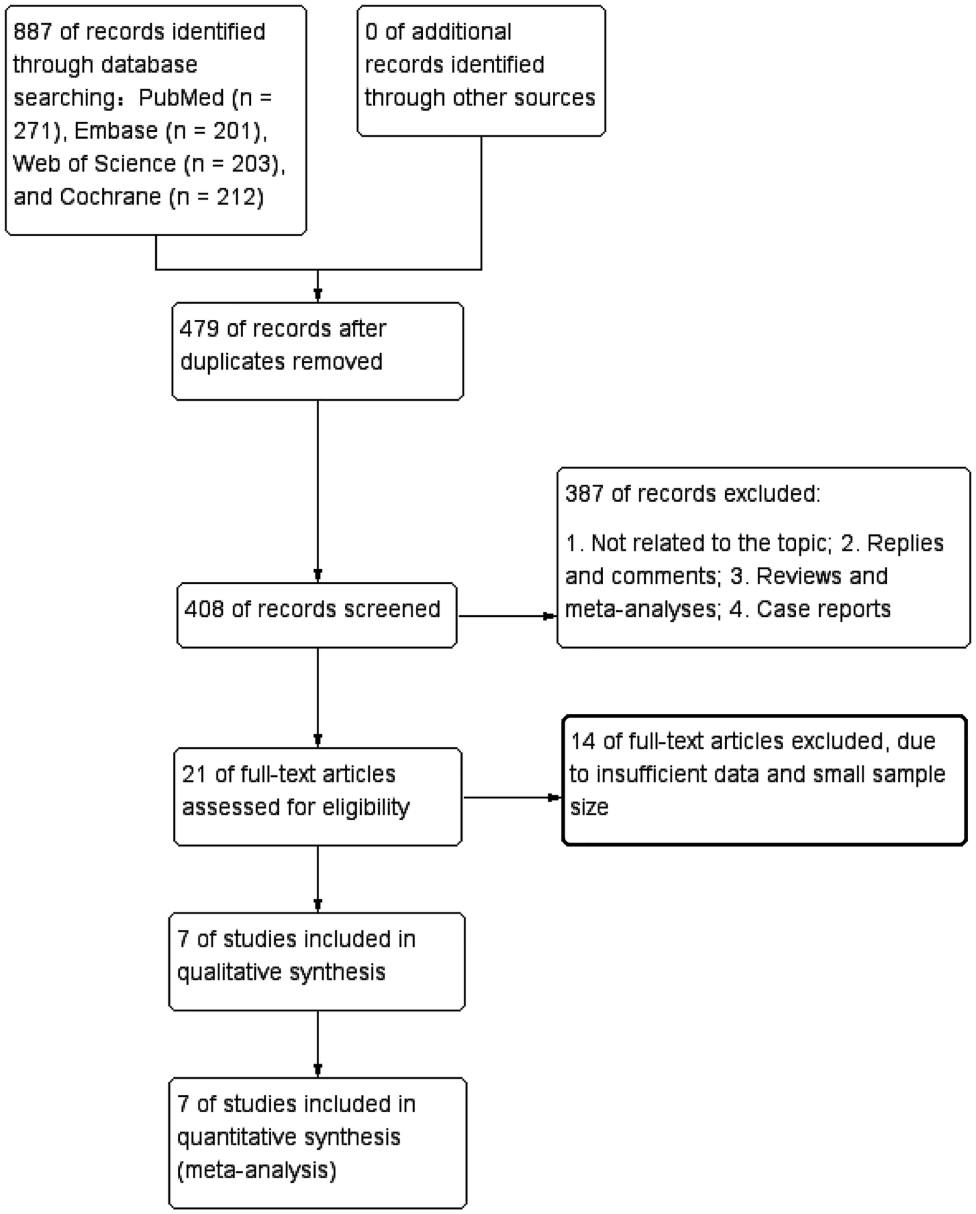
Flowchart of the systematic search and selection process.

**Figure 2:**
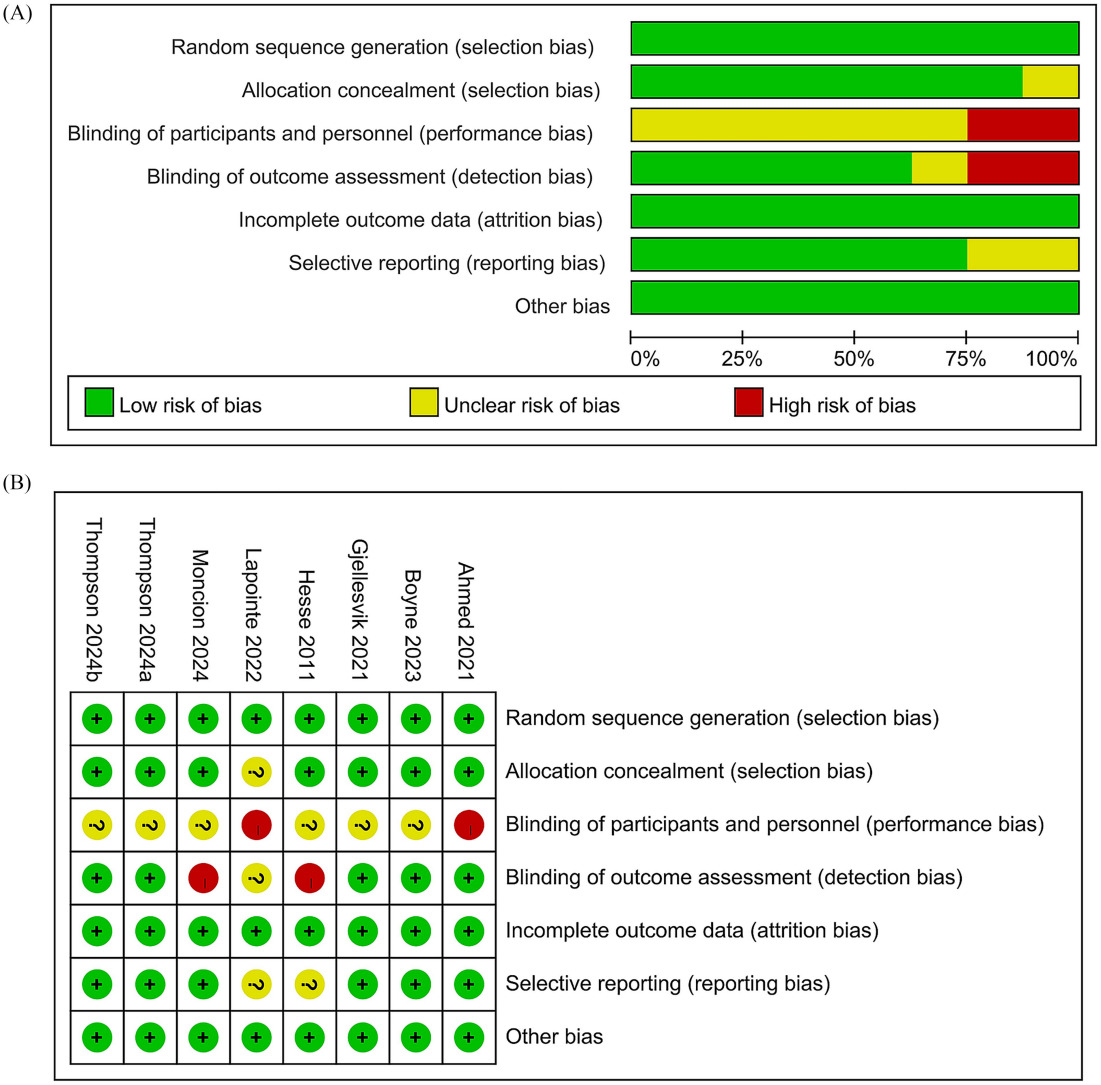
Details of the quality evaluation for included RCTs. **(A)** Risk bias of summary. **(B)** Risk bias of graph.

Figure 1 illustrates the literature retrieval and selection process. Eight hundred and eighty-seven studies were identified in PubMed (*n* = 271), Embase (*n* = 201), Web of Science (*n* = 203), and Cochrane (*n* = 212). After removing duplicates, 408 titles and abstracts were screened, and seven RCTs (18, 22–26, 31) involving 724 patients were included in the meta-analysis. Table 1 outlines the characteristics of each eligible RCT, and Figure 2 presents the quality evaluation details for all included studies. Table 2 presents the detailed high-intensity exercise protocols in each study.

**Table 1:** Characteristics of included RCTs.

Study	Study period	Country	Study design	Registration number	Intervention	Control	Sample size		Intervention time	Mean/median age		No. of male		Mean/median BMI	
							Intervention	Control		Intervention	Control	Intervention	Control	Intervention	Control
Ahmed et al. (22)	2017–2018	Pakistan	RCT	IRCT20200127046275N1	High-intensity multiplanar trunk training coupled with dual-task	Standardized trunk care regime	42	42	3 months	61.21	62.21	20	17	25.9	26.69
Boyne et al. (23)	2019–2022	USA	RCT	NCT03760016	High-intensity interval training	Moderate-intensity aerobic training	27	28	3 months	63.8	61.5	16	20	28.9	28.7
Gjellesviket al (18)	2015–2017	Norway	RCT	NCT02550015	High-intensity interval training	Standard care	36	34	8 weeks	57.6	58.7	21	20	26.9	27.8
Hesse et al. (31)	NA	Germany	RCT	NA	Intermittent high-intensity physiotherapy program	Regular physiotherapy program	25	25	12 months	62.4	61.9	13	14	NA	NA
Lapointe et al. (24)	2018	Canada	RCT	ISRCTN 17499536	High intensity interval training combined with moderate intensity continuous training	Moderate intensity continuous training	19	33	4 weeks	71.8	65.6	13	20	27.9	28.4
Moncion et al. (25)	2019–2023	Canada	RCT	NCT03614585	High-intensity interval training	Moderate-intensity continuous training	42	40	3 months	65.4	64.4	27	23	28	29.2
Thompson et al. (26)	2016–2021	USA	RCT	NCT02835313	High-intensity walking intervention	Step activity monitoring behavioral intervention	89	81	3 months	63	62	48	42	29.79	31.58
Thompson et al. (26)	2016–2021	USA	RCT	NCT02835313	High-intensity walking intervention combined with step activity monitoring behavioral intervention	Step activity monitoring behavioral intervention	80	81	3 months	62	62	44	42	30.13	31.58

**Table 2:** The high-intensity intervention protocols from the included studies.

Study	Intervention group name	Exercise type	Intensity	Frequency & duration
Boyne et al. (23)	HIIT	Treadmill/overground walking	Bursts: 30 s at max safe speed (target >60% HRR). Rest: 30–60 s	3 sessions/week, 45 min/session, for 12 weeks (total: 36 sessions)
Ahmed et al. (22)	HIMTD	Multiplanar trunk movements + dual tasks	“Heavy” on mRPE (0–10 scale). External resistance added when needed	5 sessions/week, ~38 min/session, for 12 weeks (total: ~60 sessions)
Thompson et al. (26)	FAST	Treadmill + overground walking	70–80% heart rate reserve (HRR)	3 sessions/week, ~40 min/session (30 min treadmill + 10 min overground), for 12 weeks (total: ~36 sessions)
Moncion et al. (25)	HIIT	Recumbent stepper	10 × 1-min intervals at 80–100% HRR (progressed), interspersed with 1-min recovery at 30% HRR	3 sessions/week, 19 min/session (HIIT time), for 12 weeks (total: 36 sessions)
Lapointe et al. (24)	HIIT + MICT	Upright cycle ergometer	HIIT: 30–60 s at 95% peak power output (PPO), active recovery at 40% PPO	3 sessions/week (mix of supervised HIIT & unsupervised MICT), for 24 weeks
Hesse et al. (31)	Intermittent high-intensity	Trunk/balance exercises	Not explicitly defined beyond “intensive” within sessions	Three 2-month blocks: 4 sessions/week, 30–45 min/session (total: 96 sessions over 12 months)
Gjellesviket al (18)	HIIT	Treadmill walking	4 × 4-min intervals at 85–95% peak heart rate (HR_peak_), with 3-min active recovery at 50–70% HR_peak_	3 sessions/week, for 8 weeks (total: 24 sessions)

HIIT, high-intensity interval training; HIMTD, high-intensity multiplanar trunk training + dual-task; FAST, high-intensity walking intervention; MICT, moderate-intensity continuous training; HRR, heart rate reserve; HR_peak_, peak heart rate; PPO, peak power output; mRPE, modified rating of perceived exertion (0–10 scale, where 10 is maximal); min, minutes.

### 3.2 6MWT

**Figure 3:**
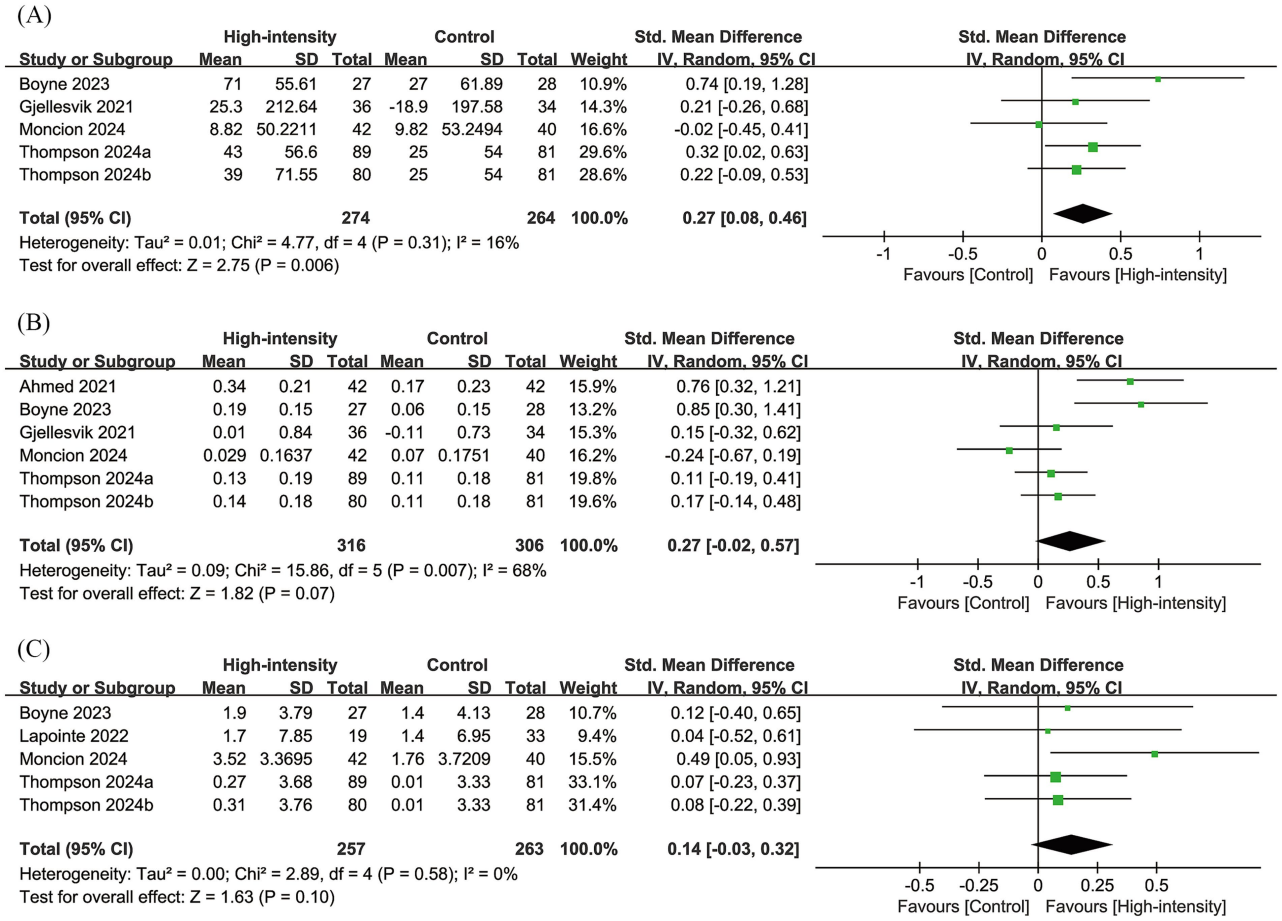
Forest plots of **(A)** 6MWT, **(B)** 10MWT, and **(C)** VO_2peak_.

Results from five RCTs with 538 individuals were pooled for the 6MWT. Meta-analysis showed a significant improvement in the 6MWT for the high-intensity exercise group (SMD: 0.27; 95% CI: 0.08, 0.46; *p* = 0.006), with no significant heterogeneity (*I*^2^ = 16%, *p* = 0.31) (Figure 3A).

### 3.3 10MWT

Results from six RCTs with 622 patients were pooled for the 10MWT. Meta-analysis showed a similar change in the 10MWT between the two groups (SMD: 0.27; 95% CI: −0.02, 0.57; *p* = 0.07), with significant heterogeneity (*I*^2^ = 68%, *p* = 0.007) (Figure 3B).

### 3.4 VO_2peak_

Results from five RCTs with 520 patients were pooled for VO_2peak_. Meta-analysis showed a similar change in VO_2peak_ (SMD: 0.14; 95% CI: −0.03, 0.32; *p* = 0.10), with no significant heterogeneity (*I*^2^ = 0%, *p* = 0.58) (Figure 3C).

### 3.5 TUG

**Figure 4:**
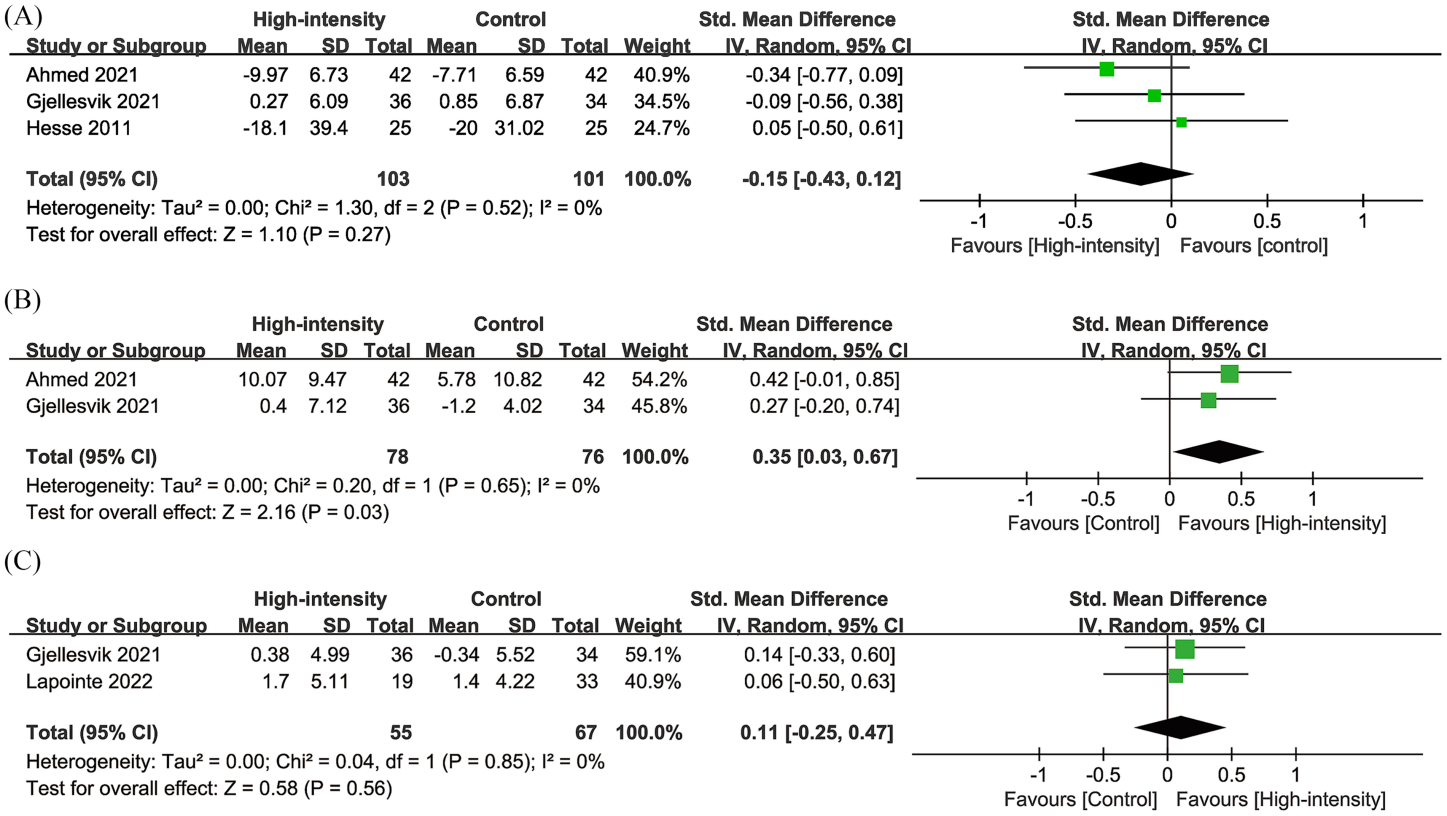
Forest plots of **(A)** TUG, **(B)** BBS, and **(C)** MoCA.

Results from three RCTs with 204 patients were pooled for TUG. Meta-analysis showed a similar change in TUG (SMD: −0.15; 95% CI: −0.43, 0.12; *p* = 0.27), with no significant heterogeneity (*I*^2^ = 0%, *p* = 0.52) (Figure 4A).

### 3.6 BBS

Results from two RCTs with 154 patients were pooled for BBS. Meta-analysis showed a significantly greater increase in BBS in the high-intensity exercise group (SMD: 0.35; 95% CI: 0.03, 0.67; *p* = 0.03), with no significant heterogeneity (*I*^2^ = 0%, *p* = 0.65) (Figure 4B).

### 3.7 MoCA

Results from two RCTs with 122 patients were pooled for MoCA. Meta-analysis showed no significant change in MoCA between the two groups (SMD: 0.11; 95% CI: −0.25, 0.47; *p* = 0.56), with no significant heterogeneity (*I*^2^ = 0%, *p* = 0.85) (Figure 4C).

### 3.8 Sensitivity analysis and publication bias

**Figure 5:**
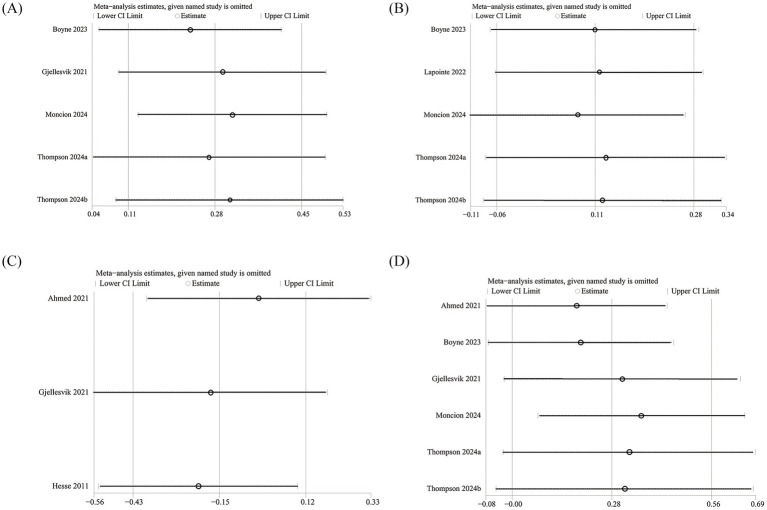
Sensitivity analysis of **(A)** 6MWT, **(B)** VO_2peak_, **(C)** TUG, and **(D)** 10MWT.

**Figure 6:**
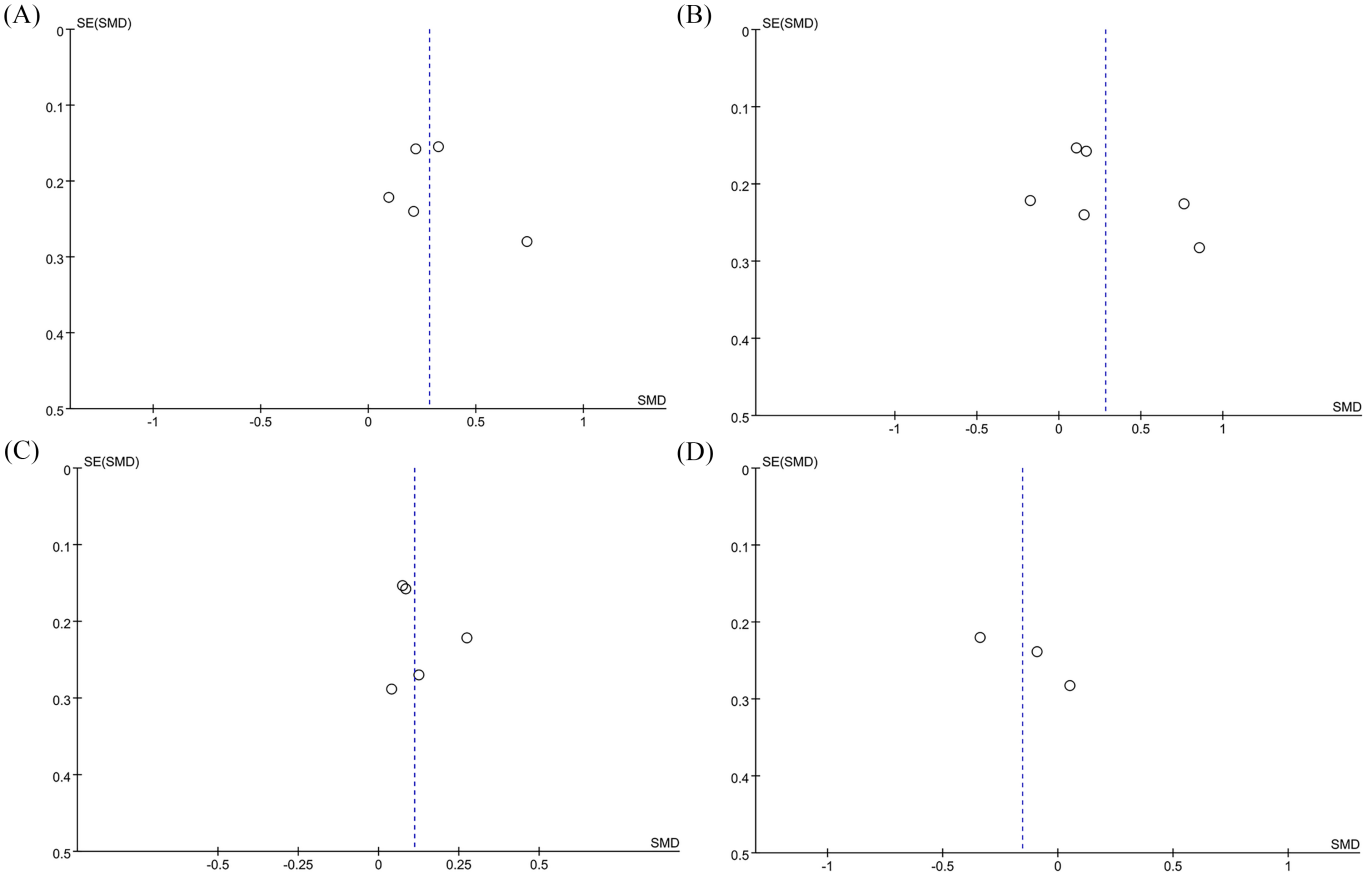
Funnel plots of **(A)** 6MWT, **(B)** 10MWT, **(C)** VO_2peak_, and **(D)** TUG.

We conducted sensitivity analyses on the 6MWT, 10MWT, VO_2peak_, and TUG results by sequentially excluding each RCT to assess its impact on the overall SMD. The sensitivity analysis showed that the total SMD remained stable after excluding individual RCTs for 6MWT (Figure 5A), VO_2peak_ (Figure 5B), and TUG (Figure 5C). However, excluding Moncion’s et al. (25) data caused the 10MWT result to shift from nonsignificant to significant (SMD: 0.36; 95% CI: 0.07, 0.66), indicating that the results are not stable enough and need to be interpreted with caution (Figure 5D). No significant asymmetry was observed in the funnel plots for 6MWT (Figure 6A), 10MWT (Figure 6B), VO_2peak_ (Figure 6C), and TUG (Figure 6D). Additionally, Egger’s tests for 6MWT (*p* = 0.543), 10MWT (*p* = 0.305), VO_2peak_ (*p* = 0.654), and TUG (*p* = 0.247) did not indicate publication bias.

## 4 Discussion

This study found that high-intensity exercise significantly improved patients’ 6MWT compared to conventional rehabilitation, with low heterogeneity. Sensitivity analysis showed no significant instability, and no potential publication bias was detected, confirming the evidence’s high quality and credibility. However, the included RCTs employed diverse high-intensity exercise protocols, as detailed in Table 2, which may contribute to the heterogeneity observed in some outcomes (notably the 10MWT) and influence the interpretation of overall effects. In addition, it should be emphasized that the conclusions of this article all indicate that there is no statistical difference compared with conventional rehabilitation. These findings suggest that adding high-intensity exercise to conventional rehabilitation can enhance stroke patients’ walking speed. Consistent with this, a meta-analysis on the effects of high-intensity interval training on post-stroke function and quality of life found that high-intensity interval training significantly improved gait speed in stroke patients (32). Despite significant heterogeneity, the meta-analysis by Baricich et al. (21) also found that high-intensity training significantly improved the 6MWT in stroke patients. Additionally, the current study suggests that high-intensity exercise may improve BBS, but due to the limited number of studies and the low quality of evidence, further research is needed to confirm this.

This study found no significant improvement in VO_2peak_ in stroke patients following high-intensity exercise, likely due to negative results reported in all included studies. This contrasts with Baricich et al. (21), who reported that high-intensity exercise significantly improved VO_2peak_ in stroke patients. However, Baricich’s et al. results showed substantial heterogeneity and may have been influenced by the small sample size effect. In contrast, no significant heterogeneity or publication bias was detected in this study, and sensitivity analysis showed no instability. The evidence quality of this study suggests that high-intensity exercise has a limited effect on VO_2peak_ in stroke patients. This negative result may be due to the fact that autonomic dysfunction in stroke patients limits the heart rate response (33). However, due to the limited number of studies, further research is needed to confirm whether high-intensity exercise can improve VO_2peak_ in stroke patients.

This study found that high-intensity exercise had no significant effect on TUG and 10MWT in stroke patients, consistent with Baricich et al. (21). Due to limitations such as small sample size and instability, more multicenter, large-sample RCTs are needed to confirm the efficacy of high-intensity exercise on TUG and 10MWT. Additionally, this study included MoCA, an unreported indicator, but found no significant effect of high-intensity exercise on MoCA in stroke patients. Although cognitive function is a key outcome for stroke survivors, the results suggest that high-intensity exercise does not improve cognitive function, which aligns with previous research.

The primary modes of exercise varied considerably across studies. Treadmill walking, either alone (18, 23, 26) or combined with overground walking (23, 26), was the most common modality. This focus on gait-specific training likely underpins the significant improvement in the walking endurance measure (6MWT). In contrast, Moncion et al. (25) utilized a recumbent stepper, Lapointe et al. (24) used an upright cycle ergometer, and Ahmed et al. (22) focused on high-intensity multiplanar trunk training with dual tasks. Hesse et al. (31) employed intensive trunk and balance exercises. These non-ambulatory or non-gait-specific modalities may partially explain the lack of significant effect on gait speed (10MWT) and the sensitivity of this outcome to the removal of the Moncion et al. study (which used a stepper).

The definition and control of “high-intensity” also differed. Most studies used percentage of heart rate reserve (HRR) as the intensity target (18, 23–26), with target intensities ranging from >60% HRR bursts (23) to 80–100% HRR intervals (18, 25). Ahmed et al. (22) relied on a modified rating of perceived exertion (mRPE) scale (targeting “heavy”), while Hesse et al. (31) described sessions as “intensive” without a specific physiological metric. Lapointe et al. (24) used peak power output (PPO) on a cycle ergometer. These variations in intensity quantification and the physiological systems primarily stressed (aerobic vs. neuromuscular) represent a source of potential heterogeneity in outcomes like VO_2peak_ and BBS.

Furthermore, the structure of the high-intensity stimulus varied. While most implemented HIIT protocols with defined work:rest ratios (e.g., Boyne: 30 s bursts; Moncion: 1-min intervals; Gjellesvik: 4-min intervals), Thompson et al. (26) and Ahmed et al. (22) prescribed continuous high-intensity walking or trunk training, respectively. Hesse et al. (31) delivered intensive intermittent sessions over a very long period. The duration of the intervention programs also spanned a wide range, from 8 weeks (18) to 24 weeks (24) and even 12 months (31), with corresponding variations in the total number of sessions (24 to 96).

This heterogeneity in exercise modality, intensity definition and target, session structure, and program duration highlights a significant challenge in synthesizing evidence for “high-intensity exercise” in stroke rehabilitation. While our analysis suggests a beneficial effect on walking endurance and balance, the inconsistent effects on other outcomes, particularly gait speed and peak oxygen consumption, may partly stem from these fundamental differences in how the intervention was applied. Future research would benefit from greater standardization in defining and reporting high-intensity parameters and matching the exercise modality more closely to the functional outcomes being targeted.

Preliminary evidence suggests that stroke patients can physiologically adapt to exercise training. However, the clinical application of exercise as a therapeutic intervention remains insufficient in the general stroke population. The decline in cardiopulmonary function after stroke may result from inadequate physical activity, leading to reduced muscle activation, impaired oxygen transport, and decreased cellular metabolism. More research is needed to fully understand the consequences and remedies of cardiopulmonary dysfunction after stroke. Systematic reviews by Luo et al. (34) and Weiner et al. (35) reported that high-intensity exercise benefits the cardiopulmonary health of stroke survivors and may be a safe and effective intervention for rehabilitation. This study confirmed that exercise training improves cardiopulmonary function in stroke patients. The mechanism may involve enhanced microvascular density, increased cardiac output, and improved mitochondrial respiration and citrate synthase activity (36). Additionally, exercise training, when added to conventional rehabilitation, can inhibit macrophage-mediated inflammatory responses in paralyzed skeletal muscles after ischemic stroke, prevent muscle atrophy, and improve physical function (37).

A meta-analysis by Machado et al. (38) on the sustained effects of high-intensity training found that stroke patients can maintain cardiopulmonary health in the short, medium, and long term after discontinuing rehabilitation. However, few studies have explored the long-term maintenance of cardiopulmonary health post-exercise, and the optimal intervention parameters for sustaining function remain unclear. Due to limited data, this study did not conduct a subgroup analysis based on training duration. Future studies should focus on longer-term follow-up to determine whether HIIT is more effective than other programs in maintaining cardiopulmonary function and whether a plateau occurs in its improvement. Clinicians should encourage stroke patients with walking function to engage in planned high-intensity training to enhance cardiopulmonary fitness. Further research is needed to assess whether stroke patients without walking function can also benefit from HIIT and to define the most suitable training model.

This meta-analysis has several limitations. First, due to the inherent limitations of exercise intervention studies, none of the seven included RCTs adequately blinded participants and personnel, which may affect the reliability of the results. Second, the RCTs varied in intervention methods (high-intensity exercise types, duration, and frequency), introducing potential heterogeneity. However, most indicators in this study showed no significant heterogeneity, minimizing its impact on the results. Third, due to the limited number of studies, we could not analyze the effects of high-intensity exercise on outcomes such as quality of life and psychological state, which requires further investigation. Additionally, data limitations prevented subgroup analysis based on intervention methods, intensity, frequency, age, race, etc., so it remains unclear whether these factors influenced the findings. Fourth, most studies were from Europe and North America (mainly the United States, Canada, and Germany), with limited data from regions such as Asia and Africa. Therefore, the global generalizability of these findings remains uncertain. Despite these limitations, this updated meta-analysis overcomes previous issues such as significant heterogeneity and small sample sizes and further supports the positive effects of high-intensity exercise on cardiopulmonary rehabilitation in stroke patients.

## 5 Conclusion

This study found that compared with usual care, high-intensity exercise significantly improves 6MWT and BBS in stroke patients, but has no significant effect on TUG, VO_2peak_, 10MWT, or MoCA. Clinicians should encourage stroke patients with walking function to engage in planned high-intensity training to enhance cardiopulmonary function. Given the limitations of this study, such as the small sample size, regional selection bias, and potential instability, future large-scale, multi-center RCTs are needed to further confirm the rehabilitation effects of high-intensity exercise on stroke patients and identify potential influencing factors.

## Data Availability

The original contributions presented in the study are included in the article/Supplementary material, further inquiries can be directed to the corresponding author.
