# Supplementary material for: Effects of high-intensity exercise on rehabilitation of patients after stroke: a systematic review and meta-analysis of randomized controlled trials with high quality
Source: Front Neurol. 2025 Aug 6;16:1565118. doi: 10.3389/fneur.2025.1565118 (PMC12366500; doi:10.3389/fneur.2025.1565118)
Supplement: Supplementary file 1 [file Data_Sheet_1.docx]

**Supplementary materials**

TableS1 Literature Search Strategy

Pubmed-271

((((("Exercise"[Mesh]) OR (((((((((((((Exercises) OR (Physical Exercise)) OR (Physical Exercises)) OR (Physical Activity)) OR (Physical Activities)) OR (Aerobic Exercise)) OR (Aerobic Exercises)) OR (Isometric Exercises)) OR (Isometric Exercise)) OR (Acute Exercise)) OR (Acute Exercises)) OR (Exercise Training)) OR (Exercise Trainings))) AND ((high-intensity) OR (high intensity))) AND (("Stroke"[Mesh]) OR (((((((((((((((((Strokes) OR (Cerebrovascular Accident)) OR (Cerebrovascular Accidents)) OR (Cerebral Stroke)) OR (Cerebral Strokes)) OR (Cerebrovascular Apoplexy)) OR (Brain Vascular Accident)) OR (Brain Vascular Accidents)) OR (Cerebrovascular Stroke)) OR (Cerebrovascular Strokes)) OR (Apoplexy)) OR (CVA)) OR (CVAs)) OR (Acute Stroke)) OR (Acute Strokes)) OR (Acute Cerebrovascular Accident)) OR (Acute Cerebrovascular Accidents)))) AND (("Rehabilitation"[Mesh]) OR (Habilitation))) AND (random*)

Embase-201


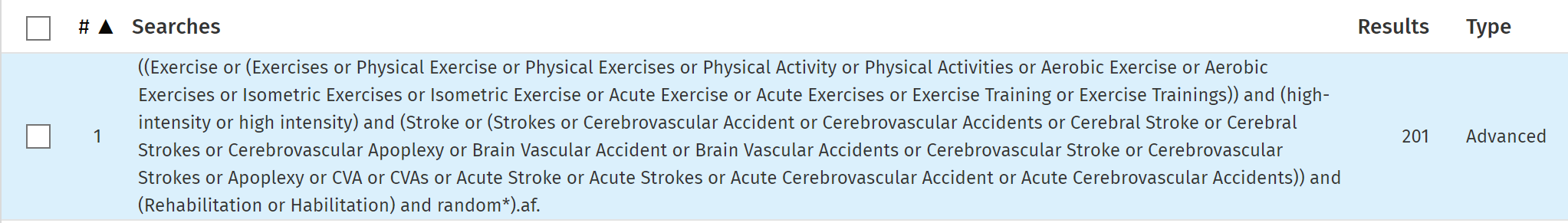


Cochrane-212


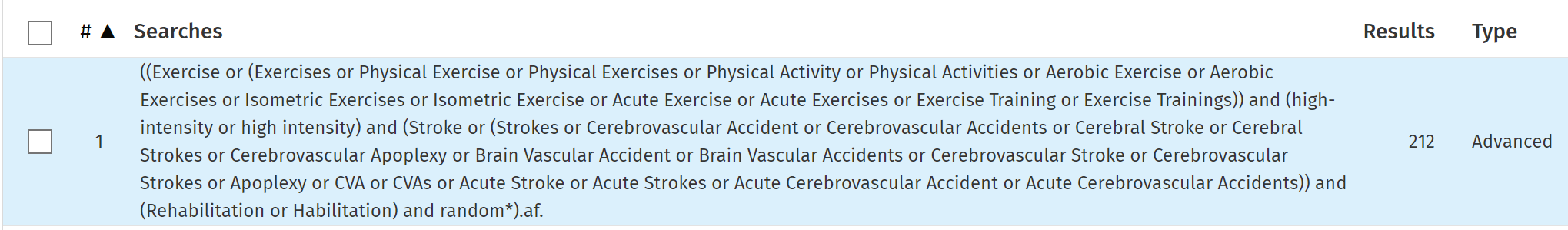


WOS-203

(((((Exercise) OR (((((((((((((Exercises) OR (Physical Exercise)) OR (Physical Exercises)) OR (Physical Activity)) OR (Physical Activities)) OR (Aerobic Exercise)) OR (Aerobic Exercises)) OR (Isometric Exercises)) OR (Isometric Exercise)) OR (Acute Exercise)) OR (Acute Exercises)) OR (Exercise Training)) OR (Exercise Trainings))) AND ((high-intensity) OR (high intensity))) AND ((Stroke) OR (((((((((((((((((Strokes) OR (Cerebrovascular Accident)) OR (Cerebrovascular Accidents)) OR (Cerebral Stroke)) OR (Cerebral Strokes)) OR (Cerebrovascular Apoplexy)) OR (Brain Vascular Accident)) OR (Brain Vascular Accidents)) OR (Cerebrovascular Stroke)) OR (Cerebrovascular Strokes)) OR (Apoplexy)) OR (CVA)) OR (CVAs)) OR (Acute Stroke)) OR (Acute Strokes)) OR (Acute Cerebrovascular Accident)) OR (Acute Cerebrovascular Accidents)))) AND ((Rehabilitation) OR (Habilitation))) AND (random*) (Topic)
